# Incentivizing an exodus: The implications of recruiting nurses from low-middle income countries to high-income countries

**DOI:** 10.1371/journal.pgph.0002355

**Published:** 2023-09-07

**Authors:** Émilie Bortolussi-Courval, Natalie Stake-Doucet, Birgit Umaigba

**Affiliations:** 1 Division of Experimental Medicine, Faculty of Medicine and Health Sciences, McGill University, Montreal, Quebec, Canada; 2 Faculty of Nursing Sciences, Université de Montréal, Montreal, Quebec, Canada; 3 Cummings School of Medicine, University of Calgary, Calgary, Alberta, Canada; McGill University, CANADA

In recent months, to palliate against a shortage of nurses, several high-income countries (HICs) have turned to low and middle-income countries (LMICs) to recruit nurses to their healthcare systems, despite the global nursing shortage disproportionately affecting LMICs [1]. This approach is ill-conceived. High-income countries do not have a shortage of registered nurses (RNs); they have a shortage of healthcare institutions providing necessary and sustainable working conditions, leading to a loss of nurses [2]. In Canada, the number of vacant RN positions increased from 10,400 to 22,400 (85.8%) from 2019–2021, despite a net growth of 7910 nurses (+2.5%) from 2020–2021 [3]. Rather than resorting to recruiting nurses overseas, governments should implement other solutions with documented success: safe nurse-patient ratios and measures to protect nurses from structural and workplace violence.

## Respect for nurses’ lives and expertise

Nurses have been fighting for safe staffing ratios for decades. The categorical refusal to listen to nurses’ clinical expertise about their own capacity to provide safe care is not only dangerous, but also a form of disrespect. For each additional patient to a nurse’s workload, the patient’s odds of dying within 30 days of admission increase by 7% [4]. In contrast, mandated staffing ratios are associated with a decrease in mortality, readmissions, length of stay and, in one study, led to savings over double the costs of their implementation [4]. Despite a growing body of evidence, these findings have rarely resulted in policy implementation; only a handful of states worldwide have implemented the required staffing ratios (Korea, Japan, Australia, four states in the United States and one province in Canada) [5].

Addressing workplace violence, which also includes sexual harassment and racism, against nurses is critical for the betterment of the working conditions at the root of the nursing retention crisis. Workplace violence is a silent but growing epidemic; in Canada, almost one third of nurses report experiencing violence at least weekly [6]. Globally, 59.2% of nurses experienced workplace violence; these events are often underreported [7]. It is associated with short staffing [8], high workloads, burnout, lowered job satisfaction and decreased retention of nurses [7]. Management’s lack of support, their singular focus on patient experiences with insufficient attention to the impact on nurses, and the nurses’ belief that reporting of violence or harassment would not lead to positive change are barriers to preventing and reducing the impact of these incidents [7]. The disregard for nurses expertise on safe staffing and nurses’ lives in regards to workplace violence is further compounded by the downward pressures on nursing wages, illustrating one of many effects of structural violence towards nurses. This trinity of disrespect of the expertise, safety, and livelihood of nurses has decimated nursing ranks.

The effects of the inertia in implementing safe ratios and protection of nurses from violence, exacerbated by the COVID-19 pandemic, are being felt; burnout rates and intentions to leave the nursing profession have never been greater. In the United States alone, since the pandemic, 100,000 (2.8%) nurses have left the bedside, and 800,000 (22.6%) more plan on leaving the profession by 2027 [6].

## Worsening equity in healthcare

Faced with nursing retention crisis, HICs are now turning to LMICs to replace nurses that have left the bedside [9, 10]. Instead of addressing the root causes, they are choosing to exploit less resourced nations with weak healthcare systems. This poses longstanding ethical problems for several reasons. First, this continuing practice will potentially deepen the maldistribution of nurses across the world; HICs have around 12 nurses per 1000 patients, and LMICs have 1 nurse per 2000 patients [10]. Around 90% of the global deficit of nurses is in LMICs [11]. Second, the true nursing shortage disproportionately affects South-East Asia, Eastern Mediterranean regions and Africa, and HICs are mostly recruiting from these regions [12]. Third, the recruitment of nurses from LMICs to HICs is leading to “brain drain” [1], defined as the depletion of health workers from LMICs, exacerbating existing health inequities. Fourth, nurses from LMICs are being recruited to work in identical conditions that pushed out existing nurses. Unsafe staffing ratios and workplace violence against nurses will simply affect a different population of nurses, but the same structural issues will remain [1]. Fifth, nurses migrating from LMICs to HICs for work are at an increased risk of exploitation, including unfair wages and stressful visa or immigration barriers [1]. This can take many forms, including but not limited to excessive demands to work overtime, lower wages than their domestically trained counterparts, threats of deportation if they do not comply with excessive demands, withholding of immigration documents, delaying payment, or the obligation of paying excessive fees if they breach their contract [1].

Recruiting nurses from LMICs fosters a de facto culture of disparity between the global north and south. High-income countries are buying into and incentivizing a perpetual cycle of high nursing turnover, thereby divesting from sustainable solutions making their own nurses *want* to join nursing or come back and *stay* at the bedside. Nurses from LMICs enter a healthcare system plagued with longstanding systemic problems, and their own experience is compounded with the systemic racism they face at every turn; from barriers to obtaining their nursing license to racist violence from patients, colleagues and management [13].

Two decades ago, healthcare researchers from South Africa, Ghana, India and Pakistan warned that healthcare systems in LMICs are “facing ultimate collapse because of the aggressive and relentless recruitment practices of [HICs]” [9]. Paradoxically, the HICs recruiting nurses from LMICs are the same nations claiming to be ‘leaders’ in global health; HICs continue to donate vaccines or medical therapies while incentivizing an exodus of nurses trained to administer them. Without clinicians, these tools are simply objects, occupying space without serving a purpose.

## Nurses are not the problem

In no way are we blaming nurses from LMICs for searching for potentially better career opportunities. We aim to underline the complacency of governments, politicians, and healthcare leaders in HICs who advertise, encourage, and depend on the recruitment of nurses from LMICs at the expense of failing to address the root causes of the current nursing retention crisis. Rather than investing in sustainable solutions to improve the working conditions of their own nurses, they are using a Band-Aid solution which has proven not only insufficient but has become a source of worldwide healthcare inequities. This is not an individual behaviour problem, but a systems problem [14].

## Change is needed now

The state of nursing care now requires all levels of governance to radically rethink how nurses provide healthcare. To do this, we call for five solutions. As nurses ourselves, we call to change the language surrounding the topic: there is no nursing shortage; there is a lack of healthcare institutions providing safe working conditions and adequate wages for nurses. A shortage implies there is not a large enough pool of available workers; nurses who chose not to work in current conditions are still part of this pool. Thus, this crisis is fundamentally a crisis of retention. Second, we call for governments and healthcare leadership of HICs to establish safe nursing ratios in all levels of care; decades of research support this central driver of nurse retention and safe, high quality nursing care [2, 4, 15]. Third, we call for these same leaders to cease unethical practices of recruitment of nurses from LMICs; the World Health Organization has developed a Code of Practice on the International Recruitment of Health Personnel, grounded in global health principles [12]. Fourth, we call for HIC leaders to develop policy facilitating the reporting of violence against nurses, including racist violence and sexual harassment, to understand the scale of the issue and to design more effective strategies protecting nurses from it. We urge them to add a metric to track the effectiveness of reporting, as reporting can lead to more harm, especially in cases of racist violence. Fifth, we also call on LMIC leaders to invest more in recruitment and retention of nurses within LMIC health systems, to address the Sustainable Development Goal of Universal Health Coverage (UHC). Nurses are critical for all countries to achieve UHC, and nurses everywhere need safe working conditions and adequate wages. If LMICs centre their efforts on the recruitment and retention of their own nurses, nurses there will be better protected against exploitation by HICs.

In conclusion, the *ad nauseam* heroization of nurses since COVID-19 seems to have allowed governments to ignore dangerous working conditions and table the implementation of safe staffing ratios and adequate wages [14]. Yet, nurses are professionals requiring decent working conditions; it is time for policymakers, governments and healthcare leaders to resolutely address the root causes of the nursing retention crisis. We are neither replaceable, nor disposable, nor heroes, nor angels.
